# Internet Video Telephony Allows Speech Reading by Deaf Individuals and Improves Speech Perception by Cochlear Implant Users

**DOI:** 10.1371/journal.pone.0054770

**Published:** 2013-01-24

**Authors:** Georgios Mantokoudis, Claudia Dähler, Patrick Dubach, Martin Kompis, Marco D. Caversaccio, Pascal Senn

**Affiliations:** University Department of Otorhinolaryngology, Head & Neck Surgery, Inselspital, Bern, Switzerland; Max Planck Institute for Human Cognitive and Brain Sciences, Germany

## Abstract

**Objective:**

To analyze speech reading through Internet video calls by profoundly hearing-impaired individuals and cochlear implant (CI) users.

**Methods:**

Speech reading skills of 14 deaf adults and 21 CI users were assessed using the Hochmair Schulz Moser (HSM) sentence test. We presented video simulations using different video resolutions (1280×720, 640×480, 320×240, 160×120 px), frame rates (30, 20, 10, 7, 5 frames per second (fps)), speech velocities (three different speakers), webcameras (Logitech Pro9000, C600 and C500) and image/sound delays (0–500 ms). All video simulations were presented with and without sound and in two screen sizes. Additionally, scores for live Skype™ video connection and live face-to-face communication were assessed.

**Results:**

Higher frame rate (>7 fps), higher camera resolution (>640×480 px) and shorter picture/sound delay (<100 ms) were associated with increased speech perception scores. Scores were strongly dependent on the speaker but were not influenced by physical properties of the camera optics or the full screen mode. There is a significant median gain of +8.5%pts (p = 0.009) in speech perception for all 21 CI-users if visual cues are additionally shown. CI users with poor open set speech perception scores (n = 11) showed the greatest benefit under combined audio-visual presentation (median speech perception +11.8%pts, p = 0.032).

**Conclusion:**

Webcameras have the potential to improve telecommunication of hearing-impaired individuals.

## Introduction

For many years the use of videophones for transmission of sign language or lip motion over telephone networks was either expensive or of low image quality, thereby limiting its use [1]. Short message service (SMS), instant messaging services or teletypewriters have therefore become the main long-distance communication modes among hearing-impaired and deaf individuals in the last two decades [2]. Written communication, however, is usually slower and less ideal to transport emotional content compared to audio-visual (AV) communication. The relative lack of long-distance communication options among hearing-impaired and deaf individuals contributes to a reduction of social connectivity and is associated with increased morbidity and mortality [3], [4]. Recently, Internet infrastructure and communication software tools have been rapidly developing and now allow both audio and audio-visual Internet communication with ever-improving quality. In comparison to conventional telephony, Internet telephony also offers broader sound frequency ranges and improved conservation of audio quality. These technical advantages of Internet over conventional telephony have translated into improved speech perception by hearing-impaired and normal hearing adults in recent, laboratory-based studies by our group [5], [6]. Earlier studies were limited to transmission of audio signals through Internet telephony, and to our knowledge, no reports on speech perception with Internet transmission of audio and visual content have been published. The current study aims to address the value of added visual content. There is evidence that cochlear implant (CI) users improve speech perception performance if visual cues are presented together with an auditory input [7]–[9]. In addition, CI users maintain their speech reading capacities after implantation [7]–[11].

Video telephony as provided by Skype™ and other Internet communication companies offer a broadband transmission of voice and image over an Internet protocol (IP) network. The Internet software sends small packets of encoded data over the Internet guided via the IP. Each data packet takes a unique pathway through the network before arriving at a receiver computer that uses the same software as the sender. The receiver’s software then collects, reconstructs and decodes all data packets before finally converting them back into an analog signal that is presented to the end-user.

Despite its potential benefits, the quality of internet video telephony transmission may be hampered by congested internet lines [12], inadequate infrastructure or insufficient bandwidth, which lead to data packet loss or delay, frame rate reduction, audio-visual asynchrony [13], or decreased signal-to-noise ratio of the video signal [14]. The web camera properties (lenses, resolution, camera software) may also influence video quality. To what extent these parameters influence speech perception, particularly by hearing-impaired individuals, has not been sufficiently addressed. Additionally, the potential of rapidly-improving Internet communication technology for helping hearing-impaired individuals remains largely unknown. The first aim of this study was therefore to test the hypothesis that current Internet technology allows sufficient transmission of lip and face motion images for adequate speech reading. The second aim was to assess the range of parameters within which visual contributions are suitable for improving communication over the Internet.

## Materials and Methods

### Ethics Statement

The study protocol was approved by the local institutional review board (Kantonale Ethikkomission Bern, Switzerland); all patients gave written informed consent.

### Test Subjects

All tests were conducted between March 2010 and July 2011 at the Department of Otorhinolaryngology-Head and Neck Surgery, Inselspital, University of Bern, Switzerland. In total, 14 deaf adults and 21 CI users participated in the study. We chose deaf individuals as a reference for the assessment of speech reading and CI users for the assessment of both speech reading and audiovisual gain. Deaf individuals were recruited from deaf community organizations (“IGGH Interessegemeinschaft Gehörlose und Hörbehinderte der Kantone Bern und Freiburg” and “proaudito, Schwerhörigenverein Bern”). Eight individuals had congenital deafness (rubella embryopathy, mumps and unknown), four had prelingual deafness (2 with meningitis, 1 with mumps and 1 unknown) and two individuals lost their hearing at the age of 22 and 44 y (progressive hearing loss). The mean age of this group was 41.6 years (range 23–63 years). All 14 deaf adults used sign language and speech reading in daily communication. They had normal vision or normal corrected vision.

CI users were recruited through a database of the audiological department of our tertiary referral center. The mean age of CI users at time of cochlear implantation was 31.3 years (range 3–63 years) and 40.6 years at the time of testing (range 18–70 years). All CI users were therefore experienced, with a mean CI-listening experience of 9.3 years (range 4–22 years). All 21 CI users had bilateral profound hearing loss, normal vision or normal corrected vision, and used an auditory-oral or total communication mode in daily life. Table 1 summarizes the clinical data of CI users included in this study. Eligible CI users had an aided minimum monosyllabic word discrimination score of 20% at 60 dB sound pressure level (SPL). Prior to testing, CI users were divided into two subgroups based on speech perception scores obtained by the HSM sentence test [15]. The subgroup of non-proficient CI users (npCI; n = 11) scored <70% correct, whereas the subgroup of proficient CI users (pCI; n = 10) scored 70% or higher in the sentence test at a SPL of 60 dB.

**Table 1:** Demographic and technical data of cochlear implanted individuals.

ID	Age		Gender	Etiology of deafness	CI-Device	Speech Processor	Communication mode
	at implantation	at test					
npCI1	4	26	F	Meningitis	Nucleus 22 Series	ESPrit 3G	Total communication*
npCI2	50	56	F	Sudden deafness	PULSARci100	OPUS2	Auditory-oral**
npCI3	45	58	F	Progressive	C40	OPUS2	Total communication
npCI4	11	25	F	Rubella embryopathy	CI24M Nucleus 24	Freedom SR	Total communication
npCI5	20	25	F	Meningitis	PULSARci100	OPUS2	Total communication
npCI6	17	30	M	Meningitis	C40	CIS PRO+	Auditory-oral
npCI7	17	22	F	Congenital	PULSARci100	OPUS2	Total communication
npCI8	61	69	M	Streptomycin	PULSARci100	OPUS2	Auditory-oral
npCI9	15	26	F	Progressive	SONATAti100	OPUS2	Auditory-oral
npCI10	52	61	F	Progressive	PULSARci100	OPUS2	Auditory-oral
npCI11	48	55	M	Progressive	HiRES90K	Auria Harmony	Auditory-oral
pCI12	21	24	F	Progressive	Freedom Implant (straight)	Freedom SP	Auditory-oral
pCI13	34	40	F	Progressive	PULSARci100	OPUS2	Auditory-oral
pCI14	41	49	F	Progressive	PULSARci100	OPUS2	Auditory-oral
pCI15	63	70	F	Progressive	C40+	OPUS2	Auditory-oral
pCI16	61	64	F	Sudden deafness	SONATAti100	OPUS2	Auditory-oral
pCI17	12	24	M	Congenital	C40+	OPUS2	Total communication
pCI18	3	24	F	Meningitis	CI22M	ESPrit 3G/N22	Total communication
pCI19	57	62	F	Meningitis	PULSARci100	OPUS2	Auditory-oral
pCI20	11	24	F	Congenital	C40+	OPUS2	Total communication
pCI21	14	18	M	Meningitis	PULSARci100	OPUS2	Auditory-oral

* Total communication includes hearing, speech reading and sign language.

** Auditory-oral communication includes hearing and speech reading.

### Speech Reading Test Procedure

We performed three series of experiments to assess speech reading cues transmitted over the Internet. A first set of experiments assessed speech reading in deaf adults under controlled conditions. Factors hypothesized to influence Internet speech communication such as different speech velocities (different speakers), camera properties (resolution, different lenses), screen properties (resolution) and Internet transmission rates (resulting in a specific frame per second [fps]) were tested by a video simulation. We presented video simulations using three different speakers (CD, 97 words/min; SF, 178 words/min; JB, 161 words/min), four screen resolutions (1280×720 px, 640×480 px, 320×240 px, 160×120 px), two screen sizes (original resolution size versus full screen mode), five frame rates (5, 7, 10, 20 and 30 fps), and three web cameras (Logitech Pro9000, Carl Zeiss lens, 2 Megapixel; Logitech C600, 2 Megapixel; Logitech C500, 1.4 Megapixel). Details about the digital generation of audio-visual video files for the simulation are shown in Text S1.

A second set of experiments tested speech reading skills in deaf individuals and CI users under real but controlled conditions (efficacy trial) by using a Skype™ Internet connection (250 kBps download and 3 kBps upload speed) between two rooms. A telephonometric communication (two persons communicate in the same room at 1 m distance) served as a reference standard. [16] This test condition is also referred as a face-to-face communication mode. Settings about a live Skype™ video transmission and its monitoring are described in Text S2.

A third set of experiments on CI users aimed to assess audiovisual cues transmitted by Internet video telephony. Auditory and combined AV stimuli with different AV-delays (0–500 ms) were used under simulated and real network conditions. Patients were asked about their overall experience with AV delay and whether they felt they required both auditory and visual stimuli or relied on either auditory or visual stimuli alone.

The German “HSM” sentence test [15] was used to assess speech perception for all test experiments across all conditions. This open set speech recognition test provides 30 lists with 20 sentences and 106 words per list. One unique list for each test condition was used to avoid learning effects. Testing was performed in a sound treated room in the free sound field using standardized equipment. Audio and video signals were delivered to the loudspeaker/laptop screen at ear level (speech signal calibrated at 60 dB SPL), at a distance of 1 m in front of the participant’s head. To balance difficulty level across subject groups and test procedures, npCI-users were tested in quiet and pCI-users were tested with simultaneous competing noise (CCITT) at a constant SPL of 50 dB for AV testing (signal-to-noise ratio (SNR) of 10 dB SPL) or 55 dB for AV-delay testing (SNR of 5 dB SPL). Subjects were asked to repeat aloud the presented words or sentences as quickly as possible or to write down. No feedback was given to the participants. Subjects were given a five-minute training session prior to testing. The percentage of correctly repeated words was used for comparison of performance across conditions. Normal hearing adults usually have a speech perception score between 90–100% in noise (SNR of 10 dB SPL) [17] [15], whereas CI users have an expected average score of 70% [18] Subjects were assigned randomly to different test sequences. To avoid order effects, different test sequences were constructed by permutation. Subjects and investigator were blinded to the different video qualities used.

All CI users were tested monaurally using the same ear throughout the entire test battery. Bilateral CI users were asked to remove the device on the poorer hearing side in order to obtain more homogenous data across subjects. The opposite ear canal was occluded with an ear plug (E.A.R. classic, Aearo Ltd., Stockport, UK) if residual low-frequency hearing was present. The specified average attenuation of these earplugs is 24.6 to 41.6 dB in the range of 250 to 4000 Hz. For the live speech reading test (Skype™ versus face-to-face) without acoustical input (visual only), CI users were required to power off their devices and to use ear plugs bilaterally.

Testing was performed in a single session with a total testing time per subject of approximately 2 hours including breaks.

### Statistics

Robust nonparametric analyses were performed to assess the potentially non-normally distributed speech perception scores from this small study population. We used the Spearman correlation test to assess the relationship between speech perception and camera properties (frame rate and resolution [megapixel]). To evaluate speech reading performance, we first used the Friedman test to identify possible differences at a 0.05 significance level in each of the following parameters: camera types, communication modes, speakers and AV-modes. Then, we used the two-tailed Wilcoxon matched pairs signed-rank test to compare groups within the parameters that were identified as different from the Friedman test. Bonferroni correction was performed for multiple testing (P_Bonf_).

## Results

### Speech Reading Performance by Deaf Adults

**Figure 1:**
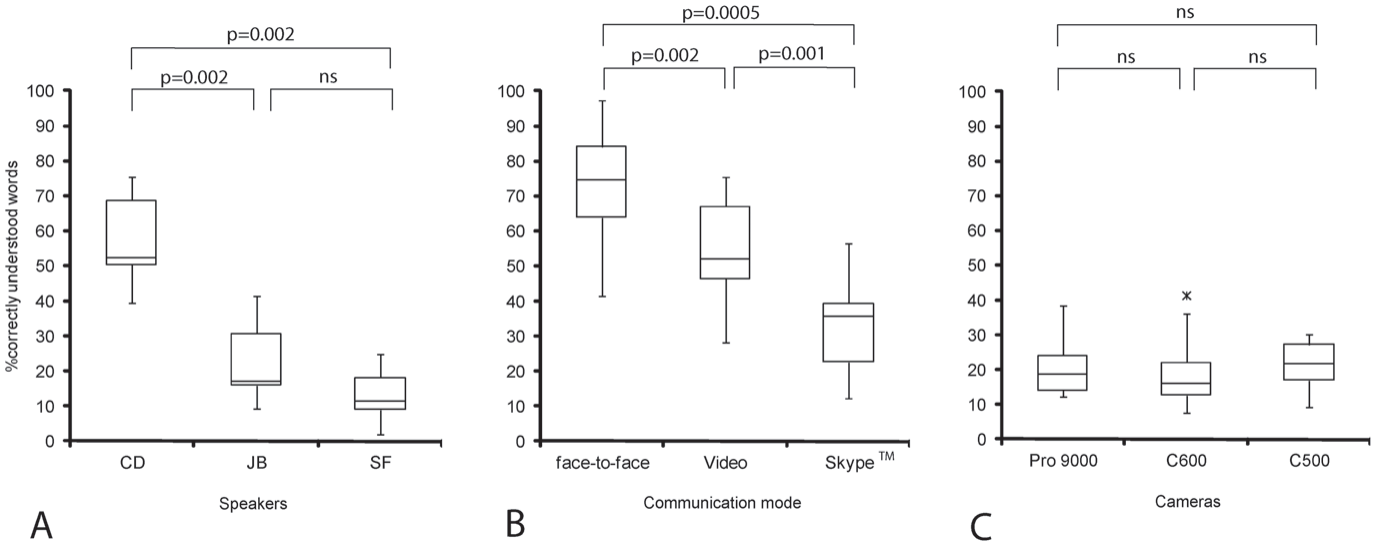
Boxplots demonstrating lower quartile, median, and upper quartile, and whiskers representing 1.5 times the interquartile range (X = outliers): Speech reading performance (correctly-repeated words in percent) from 14 deaf individuals by using (A) the same high definition web camera (Logitech Pro9000) and different speakers (CD, medical student, 97 words/s; JB, actress, 161 words/s; SF, speech therapist, 178 words/s), (B) the same speaker (CD) but different communication modes and (C) the same speaker (SF) with 3 different webcams: Logitech Pro9000, Logitech C600, and Logitech C500.

All 14 deaf adults had measurable speech reading abilities (face-to-face without sign language) with speech perception scores ranging from 41.5%–97.2% (median 74.5%). When video files were presented, scores were lower and ranged from 1.9% to 75.5% (median 51.9%), depending on the speaker. The slower speaking individual ‘CD’ (97 words/s) was better understood and this resulted in substantially higher scores (median 52.4%) compared to presentations of files by the other two speakers (median 17%, JB and 11.4%, SF, respectively). The score differences from speaker ‘CD’ to ‘JB’ and ‘SF’ were statistically significant (Figure 1A).

Similarly, the communication mode influenced speech perception by deaf individuals as shown in Figure 1B. The scores obtained with video presentation from the same speaker on a high-definition screen were statistically lower than a face-to-face communication mode (Figure 1B, p = 0.002). There was a significant loss of speech reading scores (up to 50%pts) obtained in the live Skype™ video transmission mode compared to a face-to-face communication mode (Figure 1B, p = 0.0005) or to video presentation mode (Figure 1B, p = 0.001). The median speech perception scores of deaf adults using a Skype™ transmission for speech reading alone (without using sign language) was 35.9% (range 12.3%–56.6%), which was not sufficient for satisfactory communication.

#### Camera hardware, resolution, frame rate and screen size

**Figure 2:**
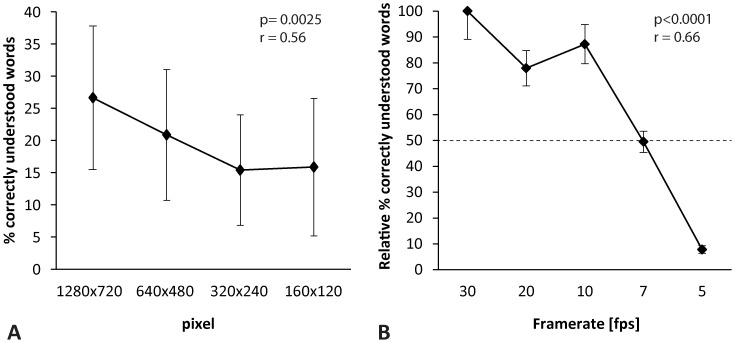
Speech reading performance (mean +/−1 SD) by n = 14 deaf individuals for 4 different spatial resolutions (A) and 5 different frame rates (B). In B, the maximum achieved speech perception at 30 fps is set to 100% (relative data). Mean speech perception scores remained above 80% until the frame rate of 10 images per second. Frame rates <10 fps were associated with a substantial reduction of the speech reading performance and frame rates at 7 fps led to a 50% reduction of the initial performance at optimal video quality. Speech reading at 5 fps was almost impossible.

The type of camera hardware did not greatly influence speech reading scores by deaf individuals; none of the comparisons across camera types reached statistical significance (Figure 1C, p = 0.79). In contrast, higher camera resolutions (Figure 2A, p = 0.0025, Spearman r = 0.56) and higher transmitted frame rates (Figure 2B, p<0.0001, Spearman r = 0.66) were associated with statistically significant higher speech reading scores. The screen size for video presentation did not greatly influence the speech reading scores. No statistical significant difference was found when comparing full screen mode vs. the video’s original size (p = 0.79, data not shown).

### Speech Reading Performance by CI-users

**Figure 3:**
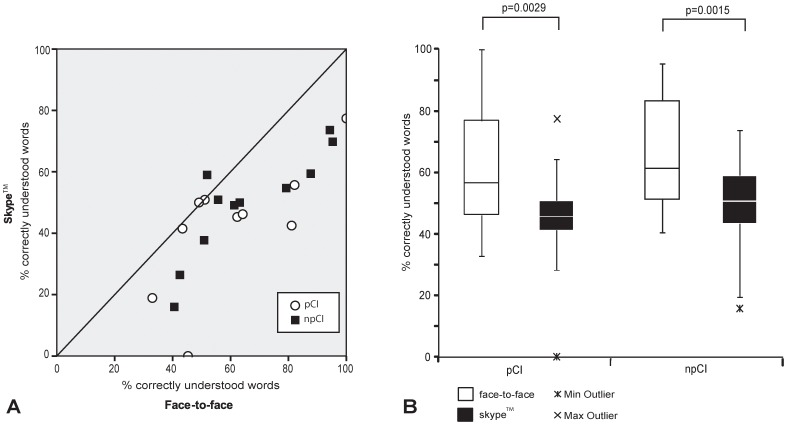
Speech reading capability of cochlear implant users. A. Comparison of speech perception scores in the absence of auditory input for n = 10 proficient (pCI) and n = 11 non-proficient (npCI) CI users for two visual communication modes (face-to-face without their implant activated vs. Skype™ video only). B. Boxplots showing speech reading scores for each condition and group.

All CI users could understand speech based on speech reading (visual only mode, Figure 3), particularly in the face-to-face communication mode. Speech perception scores were significantly lower for the Skype ™ visual only transmission mode as compared to face-to-face communication without the implant activated (pCI p = 0.0029; npCI p = 0.0015). Non-proficient (npCI) CI users were generally better speech readers compared to proficient (pCI) CI users, regardless of the communication mode (median scores of 61.3% vs. 56.7% in the face-to-face communication mode and 50.9% vs. 45.8% in the Skype™ transmission mode, figure 3).

Overall, CI users are not as good as deaf subjects at speech reading (median 61.3% vs. 74.5%), however, deaf individuals showed greater degradation of speech reading performance during a Skype™ video call (median 50.9% for npCI versus 35.9% for deaf subjects).

#### Audio-visual gain

**Figure 4:**
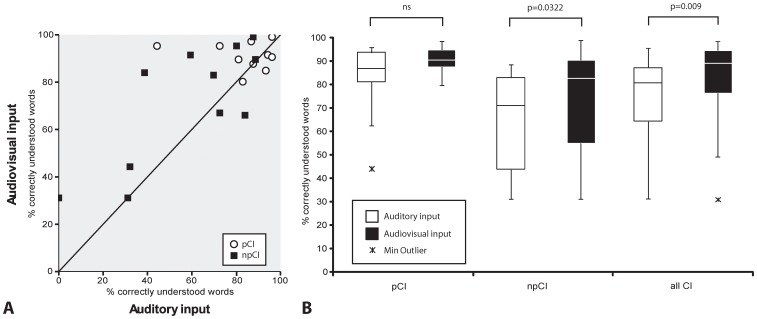
CI-users and audio-visual gain for Skype™ transmission. A. Speech perception scores of n = 10 proficient (pCI) and n = 11 non-proficient (npCI) CI users for exclusive auditory input vs. audio-visual input. B. Non-proficient CI users and the two groups combined (all CI) showed a statistically significant audio-visual gain (Boxplots). Proficient CI users showed a non-significant trend for AV-gain.

Figure 4 shows speech perception scores for audio only vs. audio-visual (AV) presentation for the Skype™ transmission mode. For all CI users (pCI and npCI combined), there is a significant overall median gain in speech perception scores of +8.5%pts (range −18% to 51%, p = 0.009) if live webcam images are added to the audio signal. NpCI users showed the greatest benefit of combined AV presentation (Figure 4). For this group, the median speech discrimination gain was +11.8%pts (−18% to 45.3%, p = 0.032) compared to the audio only presentation. A smaller, and statistically insignificant gain of +3.8%pts (−8.5 to 51%, p = 0.13) was found for the pCI-group.

#### Audio-visual asynchrony

**Figure 5:**
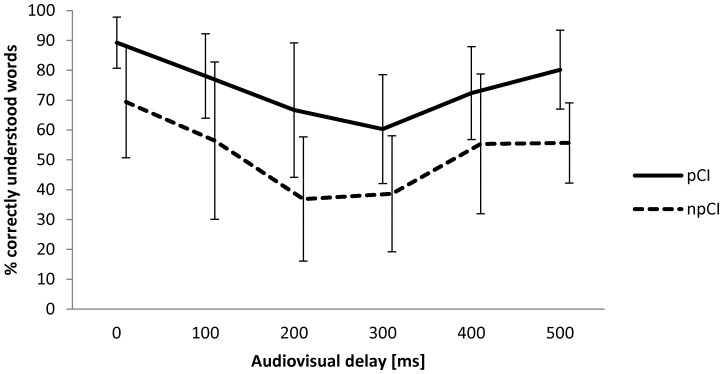
Audiovisual delay. Bimodal mean speech perception (+/−1 SD) is plotted against audio-visual delay (auditory signal proceeds image) for n = 10 proficient (pCI) and n = 11 non-proficient (npCI) CI users. Fusion of incongruent auditory and visual stimuli is not possible after 200 ms for npCI and 300 ms for pCI users. Intelligibility improved again after long AV delays because CI users did not try to fuse both incongruent signals and relied on either one of the stimuli.

Audio-visual asynchrony was associated with lower speech perception scores by CI users. The association between duration of the AV-delay and the speech perception scores are directly correlated for short delays (0–300 ms for pCI-users; 0–200 ms for npCI-users; Figure 5). Interestingly, the two groups behaved differently for longer delays, when the two signals could no longer be fused and only one stimulus could be considered for speech perception. Whereas pCI-users reported to exclusively rely on auditory signals, the npCI-users fully relied on visual stimuli. Therefore, after 300 ms and 200 ms, respectively, the speech perception scores were again higher, once the participants no longer tried to fuse the two stimuli.

## Discussion

This study demonstrates that current Internet communication technology already provides sufficient quality of video transmission for speech reading by deaf individuals. When using Internet communication, cochlear implant users show improved speech perception scores when using combined audio and visual input as compared to audio input alone. In addition, several technical parameters were identified that are associated with improved speech perception: frame rates above 15 fps, camera resolution above 640×480 px, slower speaker and shorter audio-visual delay (<100 ms). To our knowledge, this is the first study investigating speech perception using Internet video telephony technology available on the market.

### Background and Comparison with Other Studies

#### Communication mode, speaker and audiovisual gain

Speech reading is often used in conjunction with sign language or with an auditory input (AV-mode) if applicable. A multimodal communication mode increases speech perception performance by deaf and CI-using individuals. Audio-visual speech perception depends on several factors such as speech reading abilities, auditory performance and capacity of AV-fusion. CI users rely on bimodal speech comprehension, especially for face-to-face communication under noisy conditions where the auditory speech signal is degraded. Speech reading is still possible after cochlear implantation and does not change within the first postoperative year with recruiting of visual and audio-visual brain areas during communication [10].

Together with directional microphones, AV cues improve speech perception performance under adverse listening conditions [19], [20], a phenomenon which is also a known for hearing aid users [21], [22]. Enhancement of speech perception in noisy conditions by visual cues is also described for normal-hearing individuals [23] and even for deaf individuals under an exposure of multimodal congruent information [24]. Speech perception based on speech reading alone usually remains poorer in comparison to a bimodal speech information transmission, because live spoken speech transmits lip shape information at a frame rate of 25 Hz, which is 4 times lower than a pure acoustical stimulation rate [25]. Our results are consistent with the literature, in that most of the listeners experienced an audio-visual gain if congruent visual cues were transmitted, however, the two tested subgroups of CI users behaved differently. Whereas non-proficient CI users benefitted from the combined AV-mode, proficient CI users only showed a trend toward better speech perception scores. Non-proficient users showed a mean reduction of error of 26% in comparison to 3% error reduction in proficient users. One reason for this difference could be that proficient CI users experience a ceiling effect by achieving higher scores by hearing alone, leaving little room for improvement. Another reason could be a loss of speech reading skills following successful implantation; however, a longitudinal study showed that speech reading abilities remain unchanged after implantation even after several years [8]. All CI users in this study had a long listening experience ranging from 4–22 years and half of them (pCI group, n = 10) were mainly orally communicating in their daily lives. It is conceivable that pCI-users keep their compensatory speech reading skills in case they are exposed to difficult listening conditions.

Furthermore, the transmission of speech reading cues over a video screen or via Skype™ transmission led to lower speech perception scores in comparison to a face-to-face communication mode (Figures 1B and 3). There was always a loss of information observed probably because of the lack of depth of field, missed 3D perception and degradation of the signal during Internet transmission. While speech reading abilities during a live face-to-face conversation remain excellent, deaf individuals experience more speech perception difficulties during a Skype™ video call compared to CI users. It seems conceivable that deaf individuals evaluate more facial details during speech reading than CI users, who rely more on auditory signals and pay less attention to other facial cues than lip motion. In fact, it could be shown that deaf individuals have a better visual cognition compared to hearing controls [26]. Speech reading performance of our test subjects was strongly dependent on the individual speaker (Figure 1A). The factors contributing to this variability include speech velocity, lip shapes, skin, facial hair or different visual articulation, as reported earlier [25].

#### Frame rate

Foulds et al. [27] suggested a minimal frame rate of at least12 images per second for sufficient sign language transmission. For video transmission of speech reading cues, the United Nations Specialized Agency “ITU” recommended 10 years ago a frame rate of 20 fps or more [28] but with some constraints, a frame rate of 12 fps and higher could be used. A frame rate of more than 15 fps still increases speech perception performance, but to a lesser extent [22]. Theoretically, 10 phonemes per second have to be transmitted which requires a frame rate of at least 20 fps [28]. Trained and experienced lip readers, however, will achieve sufficient speech understanding by speech reading alone even under adverse network conditions with reduced frame rate (<15 fps) because of sentence reconstruction of guessed words and redundancy. Frame rates lower than 8 fps are not considered sufficient for speech reading [28]. With current Internet technology, the recommendation by the ITU-T seems to be met, since our live Skype™ video calls transmitted a mean frame rate of 15 fps (range 12–30 fps). All participants showed a benefit on speech understanding by using speech reading cues even under adverse network conditions with decreased frame rates. Speech perception was, as mentioned previously, dependant on the speaker and her speed of speaking (Figure 1A). Faster speech requires higher frame rates compared to slower speech to allow adequate transmission. The findings in this study add evidence to the strong relationship between frame rate and speech reading performance.

#### Spatial resolution and camera properties

It has been reported that communication by speech reading and sign language at a resolution of 176×144 pixels is possible despite losing many facial details [28]. The display size seems not to be the main limiting factor for speech reading [22]. Our results suggest the use of higher spatial resolutions in order to improve speech performance (Figure 2B). Video conversations at small spatial resolutions (lower than 640×480 px) should be performed in full screen mode because the lip shape information is still preserved. However, current Skype™ versions support the transmission of high definition images (720 p). Better camera properties like expensive camera lenses were not associated with better speech comprehension. Hence, even cameras affordable for a smaller budget are sufficient for audio-visual modes of speech reading.

#### Bandwidth

According to Luca De Cicco et al [29] a minimum bit rate of 40 kbps is mandatory to engage in a video Skype™ call. One decade ago, Internet communication technology did not provide sufficient bandwidth for real-time video transmission over communication networks [1]. Many attempts were made to transmit real-time video at lower bandwidths by using modern algorithms with data compression, image size reduction or intelligent recognition of hand and face movements [30]–[32]. These solutions, however, have lost importance recently with improving broadband Internet infrastructure ensuring stable and fast data connections. The latest version of Skype™ (>4.2 Beta) supports broadband transmission of high-definition video (1280×720 px), which further enhances the communication experience.

#### Signal delay

End-to-end video delay should be kept below 0.4 s [28] similar to the requirements for audio conversations in order to ensure an agreeable communication. Roundtrip time (RTT) measures the time needed for a data packet to be transmitted from the sender to the receiver plus the time back for the acknowledgment of the received packet. Current 100 MBit connections by Ethernet have normally a RTT less than 1 ms, while the RTT for wireless Internet connections (WLAN 802.11 g/n) is prolonged (<5 ms). Mean RTT for the Skype™ connection measured in this study was <1 ms (range 0–15 ms), which is an acceptable RTT length. RTT depends also on internet infrastructure and the geographical location of both sender and receiver [33].

#### Audiovisual asynchrony

AV signals are often synchronized by a form of interlaced video and audio data or by explicit AV synchronization by time-stamping [34]. Different audio and video paths can lead to a variable AV-sync delay (AV asynchrony). An incongruent AV signal is often associated with a degradation of speech perception performance. CI users have the ability to fuse incongruent auditory and visual information [11] regardless of hearing impairment or age [13], [35] which could be shown for both CI-study groups, pCI and npCI. The fusion process, however, depends on the duration of the AV-delay (Figure 5). Recent data suggests that CI users have an increased ability for cross-modal central interaction between visual and auditory processing compared to normal hearing listeners [10]. Speech perception performance of non-proficient CI users depends more on visual cues in cases of incongruent visual and auditory cues (AV conflict) [11]. Figure 5 represents this phenomenon for npCI users experiencing AV conflict (minimal speech reading performance at 200 ms intermodal delay), whereas pCI users were more resistant up to 300 ms. NpCI users reported to rely only on visual cues if unable to fuse incongruent AV information (>200 ms, Figure 5). An over-reliance on visual cues may affect speech perception performance under asynchronous AV conditions because visual stimuli could impair auditory processing based on cross-modal plasticity in cochlear implant users [36]. Therefore, a time delay between audio and image transmission over the IP-network should be kept to a minimum. Baskent and Bazo [13] demonstrated, that an intermodal delay of −108 to +203 msecs was not detectable for more than half of normal hearing test subjects. Estimates of the minimal detectable asynchrony (sound leads the image) vary widely in the literature (20 ms –150 ms) [37], however, a time window for possible AV integration of asynchronous signals ranges between 40 to 600 ms [38]. In our study, subject speech perception performance with an AV-delay of at least 100 ms fell below the performance levels of speech reading or hearing alone. The recommended acceptable time delays of up to 100 ms [28] are in line with our findings.

AV asynchrony may be related to calculation delay in the cochlear implant system, however we did not test this. Based on manufacturer data, the processing time in the implant or speech processor is negligible compared to the AV-delays occurring in video telephony.

One limitation of the presented study is the fact that only speech reading based speech perception by deaf individuals was assessed. The effects of sign language or bimodal communication (lip movements and sign language combined) on speech perception through video-transmission have not been considered. The main reason for focusing on speech reading was that all participants (deaf individuals and CI users) had some experience reading lip reading cues. Not all tested CI users were able to understand sign language. In addition, this study aimed to understand the potential of current technology for transmitting lip motion cues over the network, which is more delicate compared to hand and finger motion cues [28]. Another limitation is that our data cannot be generalized for all Internet video telephony services on the market. We have analyzed only one popular service (Skype™); nevertheless, the present study may be used as a reference for other services or similar studies, because the codecs used in Skype ™ are produced by Google’s subsidiary company On2 Technologies, the world market leader providing most modern video codecs for other Internet communication services.

### Potential Implications

Internet video telephony (in particular Skype™) offers direct communication benefit for deaf and cochlear-implanted individuals at minimal cost. We believe the four main advantages of this new technology for CI users are: 1. Bilateral hearing is possible either in free field with PC active loudspeakers or with headphones, 2. the auditory signal can be amplified up to a comfort level, while the conventional telephone is adjustable only to a limited extent, 3. broadband voice quality is near CD-quality in comparison to a low-pass filtered signal in conventional telephony [5] and 4. visual cues are available to the end-user through the web camera. The advantage of Skype™ video transmission in comparison to pre-existing videophones (based on ISDN or other networks) is the worldwide and widespread use of this free available software with more than 2.4 billion software downloads, more than 700 million registered users and more than 30 million online users. Cochlear-implanted individuals may therefore communicate with numerous normal hearing users without previous investments in communication devices. Additionally, Skype™ conference calls may be helpful for deaf individuals by using sign language interpreters. Therefore, professionals dealing with hard of hearing and deaf individuals, should recommend the use of Internet video calls for an enhanced communication experience.

### Conclusions

The present study identified several factors associated with improved speech reading performance over Internet video telephony, such as frame rates above 15 fps, camera resolution above 640×480 px, slower speaker and shorter audio-visual delay (<100 ms). Overall, Internet video telephony transmits sufficient lip shape information for speech reading by deaf and cochlear-implanted individuals. There are significant audio-visual benefits observed for CI users; however, bimodal cues with the addition of sign language for deaf individuals or auditory input for cochlear-implanted patients are still recommended for engaging in meaningful video-conversation over the web.

## Supporting Information

Text S1 **Digital generation of audio-visual video files.**
(DOC)

Text S2 **Live Skype™ transmission.**
(DOCX)
