# Supplementary material for: Internet Video Telephony Allows Speech Reading by Deaf Individuals and Improves Speech Perception by Cochlear Implant Users
Source: PLoS One. 2013 Jan 24;8(1):e54770. doi: 10.1371/journal.pone.0054770 (PMC3554620; doi:10.1371/journal.pone.0054770)
Supplement: Text S1 — Digital generation of audio-visual video files. (DOC) [file pone.0054770.s001.doc]

**Text S1. Digital generation of audio-visual video files**

Three different speakers (a speech therapist (SF), an actress (JB) and a medical student (CD)) were videotaped while reading the standardized speech test material. Location, background and ambient lighting were kept constant during filming of each speaker. Three different web cameras, positioned at 1-m distance from the speaker’s head were used for recording (Logitech Pro9000, Carl Zeiss lens, 2 Megapixel; Logitech C600, 2 Megapixel; Logitech C500, 1.4 Megapixel). The recorded digital video files (avi) served as a common basis for further processing. A video capture/processing utility for a 32-bit Windows platform (Virtual Dub version 1.9.8 [www.virtualdub.org](http://www.virtualdub.org/)) was used for video capturing and for converting the avi-video files into four different resolutions (1280x720px, 640x480px, 320x240px, 160x120px), five different frame rates (5, 7, 10, 20 and 30 frames per second [fps], 640x480px) and 5 different AV time delays (100, 200, 300, 400 and 500 milliseconds, 640x480px). All prerecorded and processed videos files were anonymized and replayed without sound, without video or with video and sound combined (AV-mode). All video and audio signals were played from a standard personal computer (Fujitsu Siemens Laptop, Amilo M1451G, Windows XP, windows media player version 11.0) connected to a commonly available active loudspeaker (Logitech, Speaker System Z320, Switzerland). A 15.4” laptop screen (1280x800px WXGA with Crystal Technology, ATI Mobility Radeon X300 graphic board) was used for displaying web camera images.
