# Supplementary material for: Internet Video Telephony Allows Speech Reading by Deaf Individuals and Improves Speech Perception by Cochlear Implant Users
Source: PLoS One. 2013 Jan 24;8(1):e54770. doi: 10.1371/journal.pone.0054770 (PMC3554620; doi:10.1371/journal.pone.0054770)
Supplement: Text S2 — Live Skype™ transmission. (DOCX) [file pone.0054770.s002.docx]

**Text S2. Live Skype^TM^ transmission**

Speech perception scores were assessed for a live test using a Skype™ audio-visual connection (DSL LAN connection 250 kBps download and 3kBps upload speed, Skype™ Version 5.3, Silk V3 codec, VP71 video-codec On2 Technologies) between 2 laptop computers (DELL Laptop Latitude E6510, USA). The hardware included a web camera (Logitech pro9000) with an integrated external microphone (2 megapixel, Carl Zeiss lenses, connected by USB 2.0). Camera resolution was set to p720 mode (1280x720 pixels). The following technical Internet connection parameters were monitored during every Skype™ call: the average packet loss was 0%, the roundtrip time (RTT) ranged from 0-15ms, the jitter ranged from 20-53 mUI (Mili Unit Interval, which reflects the percentage of time compared to the UI or one bit time) and frame rate from 12-30 fps. All live-spoken tests were conducted in two sound-proofed rooms (sender and receiver) in the free sound field calibrated at 60dB SPL. Speech loudness monitoring was used during the test. The HSM sentence test was delivered by Skype^TM^ with and without sound by a medical student (CD). The distance between subject and screen or face-to-face was 1m.
